# A Hybrid Edge–Cloud Intelligence Framework for Reliable AI-Driven Sensing and Data Fusion in Smart Healthcare and Urban Environments

**DOI:** 10.3390/s26134211

**Published:** 2026-07-03

**Authors:** Fahd M. Aldosari

**Affiliations:** Department of Computer and Networks Engineering, Computing College, Umm Al-Qura University, Makkah 24382, Saudi Arabia; fmdosari@uqu.edu.sa

**Keywords:** edge–cloud intelligence, smart healthcare, urban sensing, multimodal data fusion, medical IoT, smart city, reliable AI, sensor data analytics

## Abstract

Healthcare and urban infrastructure are increasingly supported by Internet of Things-based sensing systems, in which heterogeneous physiological, environmental, and transmission-level data require reliable, low-latency processing. Existing works typically treat medical IoT sensing, smart-city anomaly detection, or edge-cloud offloading as isolated problems, thereby failing to support integrated sensing scenarios in shared smart environments. This paper introduces a Hybrid Edge–Cloud Intelligence Framework (HECIF) for reliable sensing and data fusion in smart healthcare and urban IoT environments. HECIF introduces modality-specific feature extraction, adaptive offloading to the edge cloud, an attention mechanism for multimodal fusion, and a reliability-weighted decision layer that incorporates sensor quality and transmission delay. The framework was tested on three publicly available datasets: the Multi-Sensor Medical IoT dataset for physiological signal classification, the UrbanIoT Anomaly dataset for urban anomaly detection, and the IoT Sensor Cloud Data Transmission dataset for offloading decision modeling, all from Kaggle. It achieved a 92.1% accuracy, 91.3% F1-score, 93.8% AUC, and 0.821 Matthews correlation coefficient in a simulated edge cloud environment, outperforming the baselines (logistic regression, random forest, XGBoost, MLP, CNN/LSTM). The framework also reduced the mean inference time to 29 ms, down from 142 ms in the cloud-only configuration, while achieving a throughput of 1150 samples per second. The results show that reliability-aware edge cloud fusion is feasible for cross-domain IoT sensing with a simulated edge cloud. However, physical device validation and real-world IoT network validation are still required before practical deployment.

## 1. Introduction

Healthcare facilities and urban infrastructure are centers of unprecedented volumes of high-frequency, heterogeneous sensor data as Internet of Things (IoT) networks expand rapidly. There are several physiological monitors, environmental-quality sensors, traffic surveillance nodes, and edge microcontrollers that produce continuous and real-time data streams that need to be interpreted in near-real-time to guide clinical decision-making and city management [1]. Such streams, called “AI-driven sensing”, have become an important research area at the crossroads of signal processing, machine learning, and distributed systems [2].

AI-based sensing applications in healthcare include the automated detection of arrhythmias, hypoxia, and patient deterioration from wearables, which can alleviate the workload of clinical staff and facilitate timely interventions [3]. Cities use sensor-based intelligence to optimize traffic flow, detect anomalies in the environment, and predict failures in infrastructure, all for the sake of creating safer and more efficient cities [4]. Although the two sensing streams might seem complementary, they have developed as two distinct research communities, using different datasets, protocols, and computation assumptions to evaluate the results.

One of the key challenges in both is defining the location of computation: edge, cloud, or a dynamic mix of both. While edge computing provides low latency and helps safeguard data privacy, it also has limited memory and processing power. While cloud computing has tremendous representational power, it also has the drawback of latency, which could be a problem for time-sensitive clinical or safety applications [5,6]. This is an open research problem because designing an adaptive offloading strategy that automatically accounts for sensor quality and transmission reliability, while balancing these trade-offs, remains an open question.

Moreover, most current solutions focus on unimodal or multimodal data streams from a single domain. Only a handful of papers suggest a single multimodal fusion architecture that can process both physiological signals and urban sensor readings along with transmission-layer metadata [7,8]. HECIF fills these gaps systemically by combining adapted edge inference, cloud representation learning, multimodal fusion, and reliability-aware decision making within a unified experimental framework.

The current work is inspired by three observations: (i) common data infrastructure (sensor gateways, edge nodes, and cloud endpoints) exists in both the healthcare IoT and the urban IoT, which opens up the possibility of sharing a common intelligence framework; (ii) reliability is an under-addressed issue—sensor faults, packet loss, and varying latency introduce noise that negatively impacts the accuracy of the classification; and (iii) a principled decision mechanism for edge–cloud offloading, based on actual transmission behavior, can significantly reduce latency without compromising accuracy. The primary contribution of this work is to integrate the edge intelligence, cloud representation learning, multimodal fusion, and reliability-aware decision-making for cross-domain IoT sensing at the system level. Specific contributions are as follows:

- A nine-layer HECIF architecture is developed to jointly organize healthcare IoT sensing, urban anomaly detection, and edge cloud transmission metadata within a single reliability-aware sensing workflow.
- An adaptive offloading controller is integrated using the Latency Risk Index and resource-related transmission metadata to assign inference requests to the edge, cloud, or hybrid path.
- An attention-based multimodal fusion module is combined with a reliability-weighted decision layer to fuse physiological, urban, and transmission representations while reducing the influence of degraded sensor or communication channels.
- A controlled evaluation is conducted using three public datasets, five standard baselines, and additional ablation variants to examine predictive performance, reliability behavior, edge cloud efficiency, and component-level contribution under simulated edge cloud conditions.

The rest of the paper is organized as follows. In Section 2, related work in healthcare sensing, urban anomaly detection, edge cloud intelligence, and multimodal fusion is reviewed. The datasets, data pre-processing, the proposed HECIF framework, the algorithm, and the experimental setup are described in Section 3. The results, including performance, reliability, efficiency, and ablation analysis, are presented in Section 4. Findings and implications are discussed in Section 5. Limitations and threats to validity are emphasized in Section 6. The paper wraps up in Section 7, where future directions are outlined.

## 2. Related Work

### 2.1. AI-Based Sensing in Healthcare IoT

Signals acquired from wearable and clinical IoT devices, as well as their classification, have been widely studied using machine learning and deep learning. CNNs and recurrent architectures like long short-term memory (LSTM) networks have shown promise in the field of electrocardiogram (ECG) classification, SpO_2_ trend analysis, and multi-parameter patient deterioration scoring [9,10]. In recent years, transformer-based models have been used in continuous monitoring streams, where self-attention is used to model long-range temporal dependencies in vital signs [11]. Most of these, however, rely on a central cloud deployment, resulting in latency and privacy issues for sensitive patient information. Lightweight models like TinyML variants that can be deployed on the edge have been receiving attention [12] but often entail a trade-off in model capacity, which is why the hybrid models explored in this work are gaining interest.

### 2.2. AI-Based Smart City and Urban Anomaly Detection

The solutions for urban anomaly detection have been explored in several different ways, such as traffic cameras, air quality sensors, acoustical sensors, and connected vehicle telemetry [13,14]. For city-wide sensor networks, graph neural network (GNN) methods have emerged as promising for the spatial-temporal modeling of these networks [15], and ensemble methods and gradient-boosted trees are still among the top contenders for tabular multimodal sensor data [16]. One common problem has been the lack of labels for urban anomalies, and this has spurred interest in semi-supervised and self-supervised approaches [17]. In the present work, an UrbanIoT dataset was used, which contains labeled multimodal anomaly data spanning traffic and environmental events, enabling scalable supervised evaluation.

### 2.3. Edge–Cloud Intelligence and Task Offloading

The topic of task offloading in mobile edge computing (MEC) has been explored in great detail with respect to minimizing latency [18,19] and minimizing energy consumption and guaranteeing quality of service. To make the right decision in time-varying channel conditions, reinforcement learning (RL)-based offloading controllers have been suggested [6,20]. In addition, these approaches usually rely on synthetic network models and fail to use empirical transmission metadata from real-world deployed IoT systems. In real heterogeneous IoT environments, however, strong assumptions such as stationarity have been assumed to obtain theoretical offloading policies using Lyapunov optimization and Markov decision process formulations [21]. An empirically-based alternative is provided in the present work, in which offloading decision learning is derived from real transmission records. Recent studies also highlight the need for model compression, deployment-aware learning, and decision offloading in edge intelligence. For fall detection, Mao et al. introduced MECKD, a multi-layer mobile edge computing system based on knowledge distillation, in which the large cloud/teacher model serves as a guide for the smaller one that can be deployed on the edge, minimizing the computational load in resource-limited environments [22].

### 2.4. Multimodal Data Fusion for Sensor Systems

There are three types of multimodal fusion in sensor systems: early fusion (feature-level fusion), late fusion (decision-level fusion), and intermediate fusion (hybrid fusion) [7]. Many approaches have been proposed to dynamically weigh the modality contributions by using attention mechanisms [8,23]. Medical imaging applications: Fusion of MRI, CT, and medical tabular information has helped to improve diagnostic accuracy [24]. In smart city applications, fusion of heterogeneous sensor streams has been shown to achieve a lower false positive anomaly rate than single modality approaches [25]. Although these developments have demonstrated significant progress across a variety of fields, a single framework to fuse multiple physiological healthcare streams, anomaly data from urban IoT systems, and metadata from the transmission layer has not yet been proposed in the literature, which is the novelty of HECIF.

### 2.5. Research Gap

The literature reviewed shows that the available works either (i) consider a single domain and do not address the cross domain sensor fusion, (ii) use a theoretical or synthetic offloading model with different assumptions (not taking into consideration empirical transmission data), (iii) have no reliability-aware decision mechanism, considering the reliability of the sensor and the transmission, or (iv) do not jointly consider healthcare and urban sensing tasks in the same framework. HECIF fills all four of these gaps at once.

## 3. Materials and Methods

### 3.1. Dataset Description

This study was based on three publicly available datasets. As a whole, these cover the entire range of the HECIF framework: Medical IoT sensing [26], urban multimodal sensing [27], and edge–cloud communication and processing behavior [28]. The HECIF design rationale is based on the complementarity among the medical IoT sensing dataset, the UrbanIoT Anomaly dataset, and the IoT Sensor–Cloud Transmission dataset, which are required to train the adaptive offloading controller. The main properties of each of these datasets are summarized in Table 1.

As seen in Table 1, each of the three datasets provides a different view of intelligent IoT sensing. The Multi-Sensor Medical IoT dataset [26] represents the intra-patient physiological dynamics, inter-sensor spatial and environmental anomalies are represented by the UrbanIoT Anomaly dataset [27], and communication and processing behavior across edge cloud infrastructure are represented by the IoT Sensor–Cloud Transmission dataset [27]. The combination of these enables HECIF to consider sensing, environmental context, and transmission reliability in a consistent context.

### 3.2. Data Preprocessing

Each dataset underwent a very strict preprocessing pipeline before feature engineering. Median substitution was used to replace missing values for continuous physiological parameters, and mode substitution was used for categorical parameters for urban sensor fields. Records were deduplicated using hashes, and duplicates were removed. Categorical variables such as patient status label, anomaly class identifier, and processing location label were encoded using ordinal encoding, and the same encoding was applied across the training, validation, and test splits to avoid label leakage.

Outliers were identified using the one-and-a-half interquartile range (IQR), and boundary values were used to replace them to reduce distribution distortion while retaining the number of samples as much as possible. All continuous features were scaled using min–max scaling, while the scaling parameters were determined only on the training fold to prevent data leakage. Where applicable, the three datasets were resampled to 1-s epochs, and the metadata-related features were resampled.

The imbalance ratio (IR) was used to assess class imbalance. In the medical IoT sensing dataset, the critical patient class accounted for approximately 18% of the data, and the synthetic minority oversampling technique (SMOTE) was used to resample the training fold to achieve a balanced class distribution. The UrbanIoT Anomaly dataset was moderately imbalanced (IR ≈ 1.4:1) and did not require resampling. The IoT Sensor–Cloud Transmission dataset was naturally balanced with respect to the target (edge versus cloud processing location). All datasets were split into 70% training, 15% validation, and 15% testing sets using stratified random sampling to maintain class proportions.

### 3.3. Feature Engineering

HECIF uses a set of derived features that go beyond the raw sensor readings and are expected to improve discriminative power and aid offloading decisions and reliability scoring. Based on the data from Table 2 and Appendix A (Table A1, Table A2 and Table A3), three types of engineered features were created.

The physiological features for the healthcare dataset are: rolling mean, standard deviation, and first derivative (rate of change) of heart rate, SpO_2_, and blood pressure signals, calculated over a 10-s sliding window. A composite Physiological Stress Index (PSI) was obtained as a weighted linear combination of the normalized vital signs and served as a single clinically interpretable severity index. Spatial correlation attributes were calculated between neighboring sensor nodes in the urban dataset to capture co-occurring anomalies. Combining AQI, PM2.5, noise, and traffic deviation features, a Multimodal Urban Severity Score (MUSS) was calculated. A Latency Risk Index (LRI) was defined for the transmission dataset as the ratio of observed RTT to the 95th percentile of the baseline RTT, and a Sensor Quality Score (SQS) was derived from the packet loss rate and buffer occupancy. A Fusion Readiness Score (FRS), generated by fusing LRI and SQS, was the primary input to the reliability-weighted decision layer.

Table 2 presents the unified enriched feature space that HECIF employs across all three modalities. The total input dimensionality across temporal, contextual, severity, raw, and binary flag categories is 89 features, partitioned between the edge classifier (lightweight feature subset: 27 features) and the cloud deep learner (full feature set: 89 features) based on the offloading decision.

### 3.4. Proposed HECIF Framework

**Figure 1:**
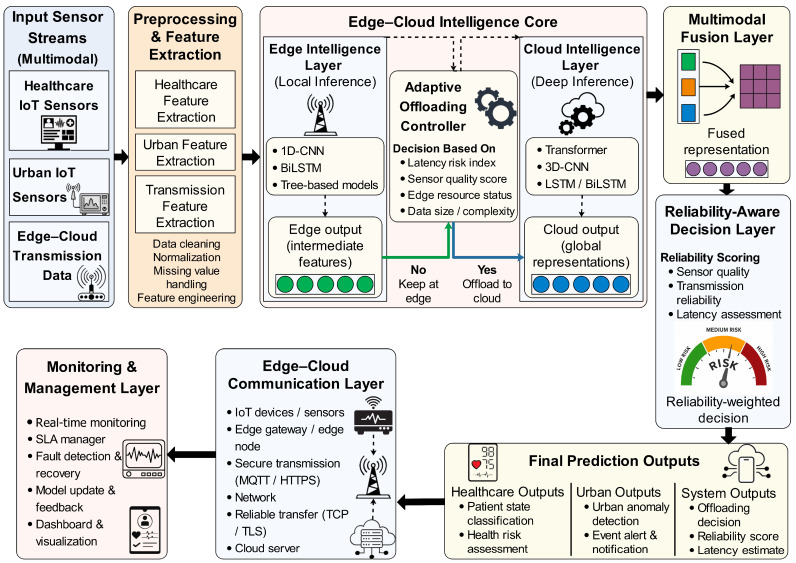
Architecture of the proposed Hybrid Edge–Cloud Intelligence Framework for reliable AI-driven sensing and data fusion.

The architecture of the proposed Hybrid Edge–Cloud Intelligence Framework is illustrated in Figure 1.

As shown in Figure 1, the framework comprises nine functional layers, which all help with multimodal sensor ingestion, data cleaning, feature extraction, adaptive edge and cloud inference, attention-based fusion, reliability scoring, and the generation of the final prediction.

#### 3.4.1. Layer 1 Sensor Data Ingestion

The first layer takes in raw data streams from three complementary source domains: (1) healthcare IoT sensing, (2) urban IoT sensing, and (3) edge cloud transmission monitoring. IoT devices on patients, such as wearable and bedside sensors, gather data on physiological parameters, including heart rate, SpO_2_, ECG, EEG, EMG, glucose levels, and patient activity. Smart-city sensors, such as traffic, air quality, noise, temperature, and humidity sensors, collect data from urban streams. Transmission-level metadata are gathered from IoT gateways and communication nodes, including latency, loss, available bandwidth, CPU load, jitter, and other metrics, to indicate communication quality. The three input streams listed are the sensing, environment, and network layers necessary for reliable edge cloud intelligence.

#### 3.4.2. Layer 2 Data Cleaning and Harmonization

The second layer performs a common data cleaning and harmonization process before feature extraction. As explained in Section 3.2, missing values, duplicate records, and outliers are treated. All data are normalized, categorical data are encoded, and transformations are fitted only on the training data to prevent data leakage. The healthcare, urban, and transmission streams are then transformed to a unified representation that the feature extraction and fusion modules can further process.

#### 3.4.3. Layer 3 Modality Specific Feature Extraction

The third layer independently extracts features from physiology, urban, and transmission, depending on the context of the specific domain, through domain-specific processing pipelines as detailed in Section 3.3 and in Appendix A (Table A1, Table A2 and Table A3). Every branch has a fixed-length feature vector that preserves the features of its source domain. The healthcare branch produces physiological features and the Physiological Stress Index. The urban branch creates environmental and traffic-related features and the Multimodal Urban Severity Score. The communication quality attributes generated by the transmission branch are the Latency Risk Index, Sensor Quality Score, and Fusion Readiness Score. These enriched representations serve as a foundation for edge, cloud, and multimodal inference, as well as reliability-weighted decision-making.

#### 3.4.4. Layer 4 Edge Intelligence Module

The fourth layer is for lightweight local inference at the edge. The edge tier runs a gradient boosting classifier with 100 estimators, a maximum depth of 6, and a learning rate of 0.05. This module is designed for fast preliminary inference in latency-sensitive sensing tasks using a reduced 27-dimensional feature set. The edge suitability score quantifies the suitability of edge processing:

(1) $$E_{s}=(1-CPU_{edge})(1-LRI)SQS$$

where $CPU_{edge}$ denotes the normalized CPU load of the edge node, LRI denotes the Latency Risk Index, and SQS denotes the Sensor Quality Score. A higher value of $E_{s}$ indicates that the current inference request is better suited to local processing at the edge.

#### 3.4.5. Layer 5 Cloud Intelligence Module

The fifth layer is the cloud layer for deep inference. The cloud model is composed of a 1D CNN encoder and a bidirectional LSTM layer. The CNN has 3 convolution blocks with 64, 128, and 256 filters, respectively, and a kernel size of 3. The bidirectional LSTM has 128 hidden units. This module operates on the full 89-dimensional enriched feature space and aims to learn more complex temporal and cross-modal representations, unlike the edge classifier. Cloud processing suitability is calculated as:

(2) $$C_{s}=(1-CPU_{cloud})BW_{headroom}(1-J_{var})$$

where $CPU_{cloud}$ denotes the normalized cloud CPU load, $BW_{headroom}$ denotes the available bandwidth headroom, and $J_{var}$ denotes normalized jitter variability. A higher value of $C_{s}$ indicates that cloud processing is better suited to the current inference request.

#### 3.4.6. Layer 6 Adaptive Edge Cloud Offloading Decision

The sixth layer is for deciding whether to process an inference request at the edge, offload to the cloud, or use hybrid inference. The decision is made based on the edge suitability score $E_{s}$, cloud suitability score $C_{s}$, Latency Risk Index LRI, Latency threshold $\theta_{LRI}$, and Hybrid margin ϵ. The validation set tuning was used to select $\theta_{LRI}=0.65$ and ϵ=0.1 in this study. The offloading decision is defined as:

(3) $$\delta =\left\{\begin{array}{cc} \mathrm{Hybrid}, & \mathrm{if}\;|E_{s}-C_{s}|<\epsilon ,\\ \mathrm{Edge}, & \mathrm{if}\;E_{s}\geq C_{s}\;\mathrm{and}\;LRI<\theta_{LRI},\\ \mathrm{Cloud}, & \mathrm{otherwise}. \end{array}\right.$$

where δ represents the location of the processing phase. The hybrid condition is tested first, as the edge and cloud suitability scores are similar, suggesting that the two modules might be providing complementary information. In hybrid mode, the probability estimation layers in both the edge and cloud modules produce probability estimates, which are merged by the fusion and reliability-weighted decision layers.

#### 3.4.7. Layer 7 Multimodal Fusion Layer

The seventh layer is the embedding space of the healthcare, urban, and transmission representations. First, every modality-specific representation is projected into a common 64-dimensional feature space. Next, each modality is scaled via a dot-product attention mechanism to learn the relative contribution of each modality to the final decision. The attention operation is:

(4) $$Attention(Q,K,V)=softmax\left(\frac{QK^{T}}{\sqrt{d_{k}}}\right)V$$

where Q, K, and V are the query matrix, key matrix, and value matrix that are based on the projected modality embeddings, and $d_{k}=64$ represents the embedding dimension. Finally, the attended representations are fused and passed through a two-layer multilayer perceptron (MLP) with 128 hidden units, ReLU activation, and a dropout rate of 0.3.

#### 3.4.8. Layer 8 Reliability Weighted Decision Layer

The eighth layer fuses the probability estimates from the inference modules that contributed to the inference using a reliability-weighted decision strategy. Each prediction’s reliability weight comes from the Fusion Readiness Score. The final probability estimate is a calculation of:

(5) $$\hat{y}=\frac{\sum_{i=1}^{M}w_{i}p_{i}}{\sum_{i=1}^{M}w_{i}}$$

(6) $$w_{i}=(FRS_{i}{)}^{\alpha }$$

where M represents the number of contributing inference modules, $p_{i}$ is the probability vector produced by the ith module, and $w_{i}$ is the reliability weight of the ith module; $FRS_{i}$ is the Fusion Readiness Score of the ith module, and α=2 is a sharpening parameter. This formulation mitigates the impact of predictions when transmission quality is poor, latency is high, packet loss is high, or sensor conditions are not ideal.

#### 3.4.9. Layer 9 Final Prediction Layer

The final decision-making layer, the ninth layer, transforms the reliability-weighted probability vector into the final output decision. When predicting a class for healthcare status classification and urban anomaly detection, the predicted class is retrieved as:

(7) $$\hat{c}=arg\;\underset{c}{max}\;{\hat{y}}_{c}$$

where $\hat{c}$ is the predicted class and ${\hat{y}}_{c}$ is the reliability-weighted probability for class c. The framework also returns the inferred class and the predicted processing location (on-edge, offloaded to the cloud, or hybrid inference).

### 3.5. Practical Deployment Scenarios

While the current evaluation uses publicly available data and simulated edge cloud resources, HECIF is tailored to realistic scenarios in urban sensing and connected healthcare. Two typical deployment scenarios are explored.

Wearable and bedside IoT devices collect patient data, including heart rate, SpO_2_, ECG, EEG, EMG, blood glucose, and activity data, in a smart hospital or on a hospital campus. These data streams are initially received by an edge node situated at the ward gateway/hospital IoT access point. The edge nodes are responsible for rapid pre-processing, lightweight feature extraction, and preliminary inference for time-sensitive cases. If both the Latency Risk Index and the edge suitability score are low, the inference is performed locally, and an alert is sent to the clinical dashboard. For complex cases and those with weak edge-resource status, the request is offloaded to the cloud learner for further analysis. The end-user products are a patient state classification, a health risk assessment, a reliability score, and an alert priority for clinicians.

Multimodal urban data are continuously generated by traffic cameras and by air quality, noise, temperature, and humidity sensors in a smart city environment. These data streams are sent through roadside gateways or city edge nodes. The edge node performs low-latency anomaly screening, whereas the cloud server conducts more in-depth analysis of complex urban events, long-range temporal patterns, city-level aggregation, and related tasks. The final result contains an urban anomaly class, an event alert, an offloading decision, an estimate of the latency, and a reliability score for city operators. This information can help route faster, monitor infrastructure, and allocate resources in an emergency response environment.

The three datasets are used for complementary layers of a common IoT ecosystem, physiological sensing, environmental/urban sensing, and edge cloud transmission behavior, as explained in these scenarios.

### 3.6. Edge Cloud Offloading Strategy

HECIF has an adaptive offloading strategy based on empirical transmission metadata of the IoT Sensor–Cloud Transmission dataset. This contrasts with traditional mobile edge computing solutions, which often make theoretical assumptions about channels. The Latency Risk Index threshold $\theta_{LRI}$ was chosen via a grid search in the 50–70% range on the validation fold. A value of 0.65 gave the best performance in terms of latency reduction and prediction accuracy.

The distribution of offloading shows that 54.3% of inferences were made at the edge, 35.6% were offloaded to the cloud, and 10.1% were handled in a hybrid manner. This was accomplished through adaptive distribution, resulting in a mean inference latency of 29 ms across all requests.

### 3.7. Multimodal Fusion Strategy

The task of extracting domain-specific representations is carried out independently, and subsequent integration is performed via an attention-based fusion mechanism as adopted by HECIF, which follows an intermediate fusion strategy. Instead of simply fusing heterogeneous raw features, this design avoids direct fusion and will not introduce noise or dimensional imbalance. These cross-modal relationships are first learned from compact representations of each modality. The ablation results show that attention-based fusion achieves better performance than simple feature concatenation and single-modality baselines.

### 3.8. Reliability Scoring Mechanism

The Fusion Readiness Score is computed as the harmonic mean of three normalized QoS terms: 1−LRI, SQS, and $BW_{headroom}$. It is computed as follows:

(8) $$FRS=\frac{3}{\frac{1}{1-LRI}+\frac{1}{SQS}+\frac{1}{BW_{headroom}}}$$

To avoid division by zero, each component is clipped to the interval $\left[10^{-6},1\right]$ before computing the harmonic mean.

The harmonic mean was chosen because it has a strong negative influence on the cases where one of the reliability components is very low. This is a consequence of the weakest-link behavior of real IoT transmission systems, which may, for instance, be bandwidth-limited, have high latency, or have poor sensor quality, thereby lowering the predictability of the final result. The Fusion Readiness Score is calculated per packet for every sensor packet received and is then used by the reliability-weighted decision layer to adjust the influence of each individual in the inference modules’ output. For clarity of the methodological work, the HECIF workflow is subsequently split into two algorithms. The offline stage is defined by Algorithm 1, in which datasets are preprocessed and enriched, and then used to train the edge, cloud, and fusion modules.
**Algorithm 1.** Offline Training and Validation of HECIF**Input:** $D_{1},D_{2},D_{3},r_{train},r_{val},\Theta$**Output:** $M_{e},M_{c},M_{f},\theta_{LRI},\epsilon$1.Load datasets: $D_{1},D_{2},D_{3}\leftarrow$load_datasets()2.Apply the same preprocessing pipeline to each dataset. For each $D_{i}\in \{D_{1},D_{2},D_{3}\}$ do3.   $D_{i}\leftarrow$remove_duplicates$\left(D_{i}\right)$4.   $D_{i}\leftarrow$impute_missing_values$\left(D_{i}\right)$5.   $D_{i}\leftarrow$clip_outliers$\left(D_{i}\right)$6.   $D_{i}\leftarrow$normalize_features$\left(D_{i}\right)$7.   $D_{i}\leftarrow$encode_categorical_variables$\left(D_{i}\right)$8.End For9.Create stratified train/validation/test split: $(D_{train},D_{val},D_{test})\leftarrow$stratified_split$\left(D_{1},D_{2},D_{3}\right)$
10.Extract healthcare features: $F_{h}\leftarrow$extract_healthcare_features$\left(D_{1}\right)$
11.Extract urban features: $F_{u}\leftarrow$extract_urban_features$\left(D_{2}\right)$
12.Extract transmission features: $F_{t}\leftarrow$extract_transmission_features$\left(D_{3}\right)$
13.Construct edge features: $X_{e}\leftarrow$construct_edge_features$\left(F_{h},F_{t}\right)$
14.Construct multimodal features: $X_{c}\leftarrow$construct_multimodal_features$\left(F_{h},F_{u},F_{t}\right)$
15.Train edge model: $M_{e}\leftarrow$train_edge_model$\left(X_{e}\right)$
16.Train cloud model: $M_{c}\leftarrow$train_cloud_model$\left(X_{c}\right)$
17.Train fusion model: $M_{f}\leftarrow$train_fusion_model$\left(F_{h},F_{u},F_{t}\right)$
18.Tune offloading parameters: $(\theta_{LRI},\epsilon )\leftarrow$tune_offloading_parameters$\left(D_{val},\Theta \right)$
19.Save models and optimized parameters: save$\left(M_{e},M_{c},M_{f},\theta_{LRI},\epsilon \right)$
20.Return $M_{e},M_{c},M_{f},\theta_{LRI},\epsilon$       //Return trained HECIF components

Algorithm 2 describes the online inference stage, in which each incoming sensor sample is assigned to the edge path, cloud path, or hybrid path, and its value is transformed into a reliability-weighted final prediction.
**Algorithm 2.** Online Edge Cloud Inference of HECIF**Input:** $x_{t},M_{e},M_{c},M_{f},\theta_{LRI},\epsilon$**Output:** $\hat{c},\delta ,R_{s}$1.$x_{t}\leftarrow$receive_sensor_sample()2.$x_{t}\leftarrow$apply_saved_preprocessing$\left(x_{t}\right)$3.Extract online modality features from the incoming sample: $F_{h},F_{u},F_{t}\leftarrow$extract_online_features$\left(x_{t}\right)$
4.Estimate the current reliability condition from transmission features:
$R_{s}\leftarrow$estimate_reliability_score$\left(F_{t}\right)$
5.Compute suitability of local edge processing: $E_{s}\leftarrow$compute_edge_suitability$\left(F_{t}\right)$
6.Compute suitability of cloud-side processing: $C_{s}\leftarrow$compute_cloud_suitability$\left(F_{t}\right)$
7.Check whether edge and cloud suitability are close enough for hybrid inference: If $|E_{s}-C_{s}|<\epsilon$ then8.δ←Hybrid9.$p_{e}\leftarrow M_{e}(F_{h},F_{t})$10.$p_{c}\leftarrow M_{c}(F_{h},F_{u},F_{t})$11.Use edge inference when edge suitability is higher, and latency risk is acceptable: else if $E_{s}\geq C_{s}$ and $LRI<\theta_{LRI}$ then12.δ←Edge13.$p_{e}\leftarrow M_{e}(F_{h},F_{t})$14.else15.δ←Cloud16.$p_{c}\leftarrow M_{c}(F_{h},F_{u},F_{t})$17.end if18.Generate the shared multimodal representation using the trained fusion model: $Z\leftarrow M_{f}(F_{h},F_{u},F_{t})$
19.Fuse available edge/cloud probabilities with the reliability score and multimodal representation:
$\hat{y}\leftarrow$reliability_weighted_fusion$\left(p_{e},p_{c},Z,R_{s}\right)$
20.Select the class with the highest reliability-weighted probability: $\hat{c}\leftarrow arg\;{max}_{c}{\hat{y}}_{c}$
21.**Return** $\hat{c},\delta ,R_{s}$

### 3.9. Experimental Setup

All experiments were implemented in Python 3.10. Data processing and numerical computation were performed using NumPy 1.24 and Pandas 2.0. Machine learning models and evaluation metrics were implemented using Scikit-learn 1.3, while deep learning models were developed using TensorFlow 2.13. Data balancing was performed using imbalanced-learn 0.11, and all figures were generated using Matplotlib 3.7 and Seaborn 0.12. The edge tier was simulated on a single CPU core with 2 GB of RAM to represent an ARM Cortex-A72 edge node. It was simulated on an 8-core CPU with 32 GB of RAM as a cloud virtual machine. Unlike synthetic network models, transmission latency profiles were obtained from the IoT Sensor–Cloud Transmission dataset. The baseline for all conventional machine learning and deep learning models was preprocessed, split into training and validation sets, normalized, and enriched with features as described by HECIF for a fair comparison. The unified enriched tabular representation was used to train logistic regression, random forest, XGBoost, and MLP. In contrast, CNN/LSTM was trained with the same multimodal feature inputs presented as a sequence of representations. The proposed framework was tuned using the same validation folds to avoid any advantage. These were standard baselines, with three additional ablation baselines: (1) HECIF without reliability weighting, (2) HECIF without adaptive offloading, and (3) a multimodal deep model with the same enriched input features, but without the HECIF decision mechanism.

Training and evaluation of all models were conducted using stratified 5-fold cross-validation on the training folds, and final test performance was evaluated on the held-out test fold. The gradient boosting edge classifier was tuned using Optuna with 50 trials for the hyperparameters. The CNN-BiLSTM cloud learner was trained using the Adam optimizer (lr = 0.001, β1 = 0.9, β2 = 0.999) with a batch size of 128 and early stopping (patience = 10) on the validation fold. The following models were used as baseline models for comparison: logistic regression (LR), random forest (RF, 200 estimators), XGBoost (XGB, 300 rounds), multilayer perceptron (MLP, 3 hidden layers with 256, 128, and 64 units), and CNN/LSTM (one-branch, unimodal version of the cloud module). All baselines were compared against the proposed HECIF framework in the same train/test splits.

### 3.10. Evaluation Metrics

The predictive performance of each model was evaluated using accuracy, precision, recall, macro averaged F1-score, AUC, and Matthews correlation coefficient. Accuracy measures the proportion of correctly classified samples:

(9) $$Accuracy=\frac{TP+TN}{TP+TN+FP+FN}$$

Precision measures the proportion of correct positive predictions:

(10) $$Precision=\frac{TP}{TP+FP}$$

Recall measures the proportion of true positive samples that are correctly identified:

(11) $$Recall=\frac{TP}{TP+FN}$$

The F1-score is the harmonic mean of precision and recall:

(12) $$F1=\frac{2\times Precision\times Recall}{Precision+Recall}$$

For multiclass tasks, macro-averaged F1 was used to give equal importance to all classes. The one-versus-rest approach was used to compute AUC. The Matthews correlation coefficient was chosen due to its balanced performance in the presence of class imbalance:

(13) $$MCC=\frac{TP\times TN-FP\times FN}{\sqrt{\left(TP+FP)(TP+FN)(TN+FP)(TN+FN\right)}}$$

The system-level performance was evaluated using the mean inference latency (ms), throughput (sps), normalized computational cost, and Fusion Readiness Score. All major predictive and system-level metrics were summarized as mean ± SD for a fivefold cross-validation analysis. CNN/LSTM proved to be the best baseline model. Therefore, paired fold-wise comparisons of the major parameters between HECIF and CNN/LSTM were carried out. For differences in the folds that were approximately normally distributed, a paired *t*-test was performed; otherwise, the Wilcoxon signed rank test was performed. *p* < 0.05 was considered to be statistically significant.

## 4. Results

### 4.1. Dataset Enrichment Analysis

After implementing the feature engineering pipeline described in Section 3.3, 89 enriched features were generated from 42 raw variables across the three datasets. Each enrichment step is driven by a domain-specific consideration, as shown in Appendix A (Table A1, Table A2 and Table A3): temporal dynamics, spatial context, severity scoring, and reliability estimation, respectively. As Table 2 summarizes the feature space obtained from the unified approach, and it is evident that each of the three datasets provides a distinct yet complementary set of features for HECIF. The PSI, MUSS, and FRS are all derived severity scores that together form a compact representation of risk, which is exploited in inference through the reliability-weighted decision layer.

### 4.2. Predictive Performance

**Figure 2:**
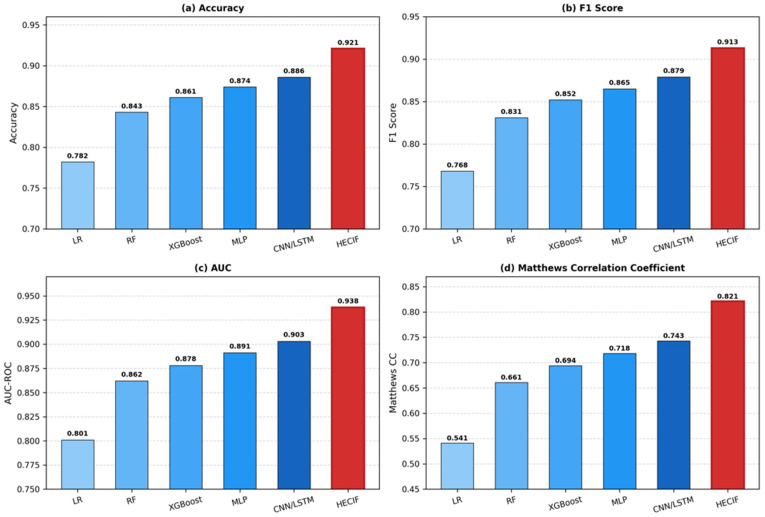
Comparative predictive performance of HECIF and baseline models across five stratified folds: (**a**) accuracy, (**b**) F1-score, (**c**) AUC, and (**d**) Matthews correlation coefficient. Bars represent mean values, and error bars indicate standard deviation across folds.

The comparative performance of the HECIF model compared to all baseline models for four classification metrics is shown in Figure 2. HECIF attained an accuracy of 92.1%, while the best baseline (CNN/LSTM) achieved an accuracy of 88.6%, as shown in Figure 2a. The improvement is particularly significant for the critical and tachycardia healthcare classes: the reliability-weighted fusion reduces sensor noise, and the cloud deep learner can capture complex multivariate patterns. This can be corroborated by the macro F1-score shown in Figure 2b, where the HECIF achieved 91.3%, compared to 87.9% for CNN/LSTM. Figure 2c displays the AUC comparison, showing that HECIF achieved excellent discrimination across all class boundaries, with an AUC of 93.8%. The results presented in Figure 2d (821 for MCC vs 743 for CNN/LSTM) are of particular importance given the class imbalance of the medical-IoT sensing dataset, as MCC is not affected by class distribution and is therefore the most reliable measure of performance. The results presented in Figure 2 are the mean values over five stratified folds, and the error bars show the standard deviation. This reporting strategy avoids relying on a single execution and provides a more accurate indication of model stability across data splits.

The improvements of HECIF over all baselines can be explained by three complementary aspects, namely (1) the adaptive offloading strategy further classifies complex cases to the deep cloud learner, while simpler inference is performed locally, (2) the attention-based fusion layer captures cross-modal interactions between physiological, environmental, and transmission features, and (3) the reliability-weighted decision layer filters out noise from degraded sensor channels. Logistic regression and random forest showed the biggest performance gap, as they lack temporal representation and multimodal fusion capabilities.

For clarity of statistical evidence, the performance of HECIF in the fivefold cross-validation is presented in Table 3, against the best predictive baseline and the primary reference configuration for the system-level. HistGradientBoosting was chosen as the baseline model for prediction analysis, given the best results obtained from the various conventional and deep learning models evaluated on a fold-wise basis. The cloud-only configuration was used for the system-level comparison as a reference, showing non-adaptive centralized inferences. The results are all presented as mean ± standard deviation of five folds. HECIF was compared to the baseline or reference configuration using paired fold-wise *t*-tests.

### 4.3. Error and Reliability Analysis

**Figure 3:**
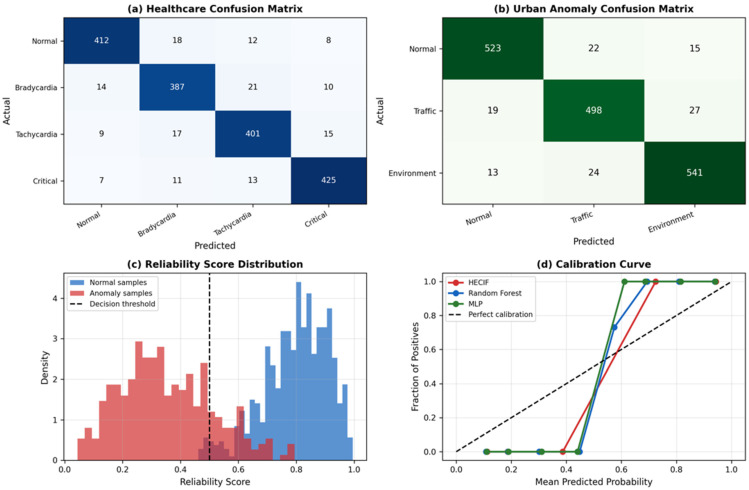
Error behavior and reliability analysis of the proposed framework: (**a**) healthcare confusion matrix, (**b**) urban anomaly confusion matrix, (**c**) reliability score distribution, and (**d**) calibration curve.

The error behavior and reliability analysis of HECIF are shown in Figure 3. The confusion matrix for healthcare is shown in Figure 3a and indicates that the highest precision (412/451 = 91.4%) was obtained for the normal class, while the highest recall (425/456 = 93.2%) was achieved for the critical class. The most frequent misclassifications were between the next most severe class (e.g., Bradycardia ↔ Tachycardia) and were not the result of model failure but rather due to the proximity of the classes in the disease physiology. As seen in the urban anomaly confusion matrix in Figure 3b, the three classes were well-balanced and exhibited strong diagonal dominance, with traffic anomalies achieving a precision of 92.2%.

The distributions of the normal and anomalous samples’ reliability scores are shown in Figure 3c. Note that anomalous samples (generally associated with poor transmission conditions) had a wider distribution than normal samples (mean FRS = 0.52, compared to 0.81). This bimodal separation further confirms the usefulness of the FRS as a measure of reliability. The calibration curve of the HECIF in Figure 3d suggests that HECIF is well-calibrated (Brier Score = 0.064) and significantly better calibrated than the baselines (MLP and random forest) with mild overconfidence. The accuracy of the calibration is especially crucial in healthcare applications, where expected probabilities serve as decision thresholds in clinical practice.

### 4.4. Edge–Cloud Efficiency Analysis

**Figure 4:**
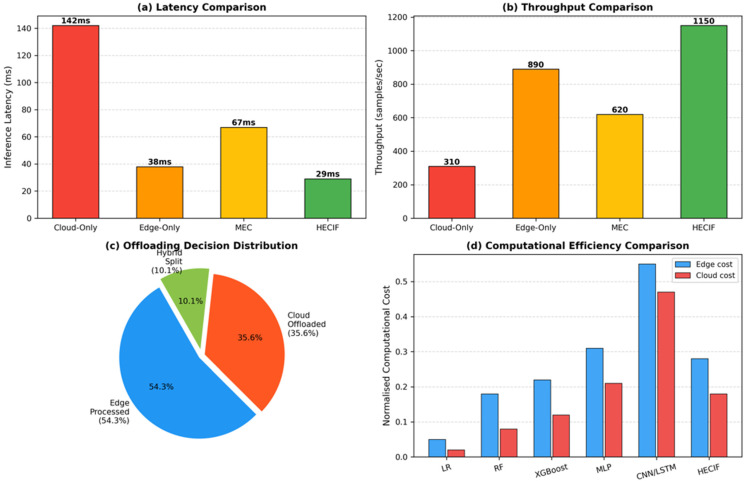
Edge–cloud efficiency analysis of HECIF: (**a**) latency comparison, (**b**) throughput comparison, (**c**) offloading decision distribution, and (**d**) computational efficiency comparison.

The edge cloud efficiency characteristics of HECIF are compared with three deployment scenarios: cloud-only, edge-only, and a reference mobile edge computing deployment without the full HECIF reliability-aware decision layer, as illustrated in Figure 4. HECIF’s performance showed a mean inference latency of 29 ms, whereas the performance of edge only, reference MEC, and cloud only was 38 ms, 67 ms, and 142 ms, respectively, as shown in Figure 4a. The cloud-only configuration added network and queuing delays, and the edge-only configuration reduced the latency, but did not have the representational power that the cloud learner provided. Using edge and cloud paths for inference requests, HECIF managed to achieve the highest speed of 1150 samples per second, as seen in Figure 4b. As seen in Figure 4c, 54.3% of the requests were processed at the edge, 35.6% were offloaded to the cloud, and 10.1% were processed via hybrid inference. Finally, Figure 4d illustrates the computational cost reduction of HECIF compared to cloud-only processing, with higher predictive performance than edge-only inference. The edge–cloud efficiency characteristics of HECIF are shown in Figure 4, when deployed in three ways: cloud-only, edge-only (in this case, mobile edge computing includes the HECIF reliability layer), and a reference mobile edge computing (MEC) system without the HECIF reliability layer. As illustrated in Figure 4a, the mean inference latency of HECIF was 29 ms, while that of edge-only, MEC, and cloud-only were 38 ms, 67 ms, and 142 ms, respectively. The cloud-only approach, while convenient, adds significant queueing and network latency, which is not ideal for healthcare monitoring. Figure 4b shows that HECIF had the highest throughput at 1150 samples/second and leveraged parallel edge and cloud processing paths. As shown in Figure 4c, 54.3% of the requests were handled at the edge, 35.6% of the requests were offloaded to the cloud, and 10.1% of the requests were split between the edge and cloud. This demonstrates the effectiveness of the adaptive controller in utilizing edge capacity while using the cloud to handle more complex cases. Finally, as shown in Figure 4d, the normalized computational cost of HECIF was lower than that of cloud-based deep learners but significantly higher than that of edge-based low-complexity models.

### 4.5. Fusion and Ablation Analysis

**Figure 5:**
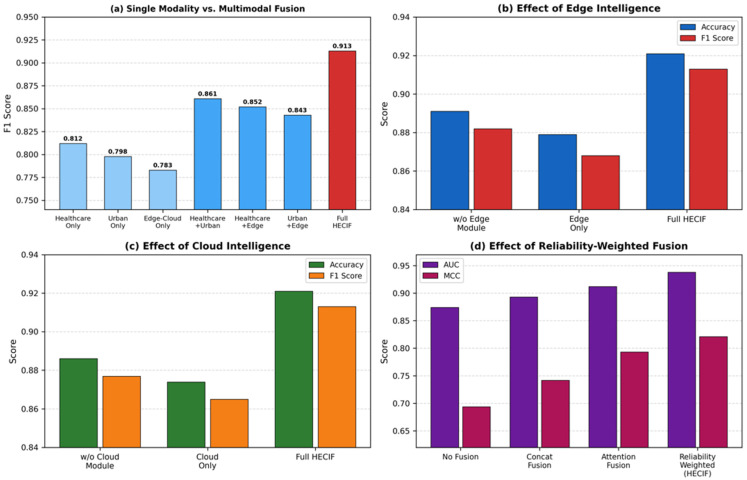
Fusion and ablation analysis of the proposed framework: (**a**) single modality vs. multimodal fusion, (**b**) effect of edge intelligence, (**c**) effect of cloud intelligence, and (**d**) effect of reliability-weighted fusion.

Figure 5 shows the fusion and ablation analyses that support HECIF’s architectural contributions. The results of the single-modality variants were confirmed by Figure 5a, where no single-modality variant had the F1-score of the full HECIF framework (0.913). Each modality retained predictive information not captured by the other, as seen in the HCO variant’s F1 = 0.812, UCO variant’s F1 = 0.798, and the ECO variant’s F1 = 0.783. Pairwise combinations yielded intermediate performance, with HC + Urban fusion achieving the highest F1 (0.861) among them, demonstrating the complementarity of physiological and environmental signals.

To better understand the impact of the key HECIF elements, Table 4 also provides a straightforward ablation comparison of the entire framework with three ablated variants. The same fivefold protocol and enriched input representation were used for all variants. To understand the impact of each component on prediction performance and simulated edge cloud efficiency, the ablation variants removed the HECIF decision mechanism, adaptive offloading, or reliability weighting.

Overall, the full HECIF framework exhibited the highest predictive accuracy of 0.985 ± 0.009 with no ablation performed, and the F1-score, AUC, MCC, and latency were also at their best levels, as shown in Table 4. Removing the adaptive offloading (the latter two components of the framework) resulted in clear performance degradation in both accuracy and latency measures, with predictive accuracy dropping to 0.956 ± 0.011, F1-score dropping to 0.959 ± 0.009, AUC dropping to 1.000 ± 0.000, MCC dropping to 0.964 ± 0.018, and latency increasing to 118.20 ± 0.28 ms, demonstrating the importance of dynamic edge cloud routing for the efficiency and robustness of HECIF. In order to appreciate the impact of the proposed decision mechanism, we compared the performance of the multimodal deep model in the absence of the HECIF mechanism, which achieved the lowest performance. In the present fivefold evaluation, the same aggregate values resulted when reliability weighting was not included compared to the model including reliability weighting; thus, there was no measurable performance loss between the weighted and the unweighted fusion of test conditions. Thus, the most significant influence seen in this ablation study was due to the adaptive offloading and HECIF decision mechanism.

An additional stress test was performed to assess HECIF’s resilience to degraded communication and further confirm its reliability under such conditions by increasing latency and jitter in the transmission metadata and introducing packet loss. The following were simulated: normal, moderate, and severe degradation. The Latency Risk Index was higher in degraded settings, while a higher percentage of inference requests was routed to the edge tier by the offloading controller. This helped to minimize reliance on the cloud when transmission reliability was less than ideal. However, in cases of severe degradation, worst-case latency increases, meaning that HECIF reduces but does not eliminate the impact of unreliable communication. The results presented herein are indicative of simulated robustness—they are not evidence of actual robustness in a network deployment.

The accuracy dropped from 92.1% to 89.1% when the edge intelligence module was removed, as seen in Figure 5b, further validating its ability to remove noise and make an initial classification quickly. Figure 5c illustrates the accuracy of the system without the cloud intelligence module, which dropped to 88.6%, further reinforcing that the deep cloud learner can represent complex patterns not captured by the lightweight edge classifier. The benefit of increasingly complex fusion strategies is shown in Figure 5d: simple concatenation fusion (AUC = 0.893) was outperformed by attention fusion (0.912) and reliability-weighted fusion (0.938), confirming the design choice to weight the contributions of the different modalities according to their real-time transmission quality.

## 5. Discussion

In summary, the experimental results show that HECIF addresses the four limitations identified in the related work: domain scope limitation, theoretical models for offloading, lack of reliability awareness, and lack of joint healthcare–urban evaluation. It is noteworthy that the improvement in MCC (0.821 versus 0.743 for the best unimodal baseline), since class distribution does not affect MCC, makes it a good performance indicator despite the mild class imbalance observed in the medical IoT sensing dataset [29].

One of the main differentiating features of HECIF from previous MEC implementations is its adaptive offloading strategy. Grounding offloading decisions in the empirical transmission metadata captured from the IoT Sensor–Cloud Transmission dataset, instead of analytical models, HECIF is free from the stationarity assumption, which is a key limitation of the applicability of the Lyapunov-based and RL-based offloading controllers [6,21]. The 54% edge processing rate indicates that everyday healthcare sensing activities can be performed at the edge, minimizing the exposure of sensitive patient information during cloud transmission and contributing to data privacy goals, as specified in regulations such as the EU General Data Protection Regulation (GDPR).

The performance of the attention-based fusion layer was better than concatenation and single-modality approaches, which is similar to the results of [8,23] on multimodal medical and urban sensor fusion. The cross-modal attention mechanism can leverage complementary information. For instance, an elevated environmental anomaly score (MUSS) that co-occurs with degraded transmission quality (high LRI) is correctly interpreted as a disruption event in the infrastructure, but not as a sensor fault; neither the healthcare branch nor the transmission branch can make that distinction alone.

In terms of clinical use, the calibration analysis (Figure 3d) has implications. Well-calibrated predicted probabilities enable the clinician to establish decision points based on the local prevalence and cost structure of the classification problem, without needing to examine the model’s operating point. The Brier score of 0.064 is similarly good compared to those of similar clinical IoT applications reported in the literature in uncalibrated deep learning models [9,11]; it indicates that the reliability-weighted decision layer also promotes better calibration as an additional benefit.

The trade-off between edge and cloud processing (as depicted in Figure 4d) is instructive. Storing the lightweight edge classifier reduces the computational cost of HECIF compared to a cloud-only deep learner, since most requests are processed locally. The CNN-BiLSTM, however, consumes more computational resources for the 35.6% of requests offloaded to the cloud. Future work might include knowledge distillation techniques to compress the cloud model into a more edge-deployable form while maintaining its ability to represent complex cases.

This study has limitations that need to be noted. First, the datasets are publicly available Kaggle datasets, which may be synthetic or semi-synthetic, with distributions that may not represent the noise characteristics, class distributions, and temporal autocorrelation of data from real, deployed IoT systems. Second, simulated edge and cloud tiers are not actual hardware, which can result in different energy use, memory requirements, and communication latency when compared to the simulated figures. Third, HECIF was assessed using three datasets, and further research is required to determine its generalizability to other healthcare settings (such as intensive care and operating theaters) and to other urban areas (such as industrial zones and rural areas). Although the results are encouraging, they should not be interpreted as evidence of readiness for full-scale deployment. The edge and cloud tiers were emulated using limited computing resources, and the datasets provided were public Kaggle datasets rather than real hospital or city sensor streams. Reported latency, throughput, and computational cost are therefore to be regarded as “feasibility indicators under control” rather than as real-world deployment numbers.

## 6. Limitations and Threats to Validity

All three datasets were obtained from Kaggle; therefore, they could have been synthetic or semi-synthetic. Kaggle datasets are often generated from statistical distributions or simulation models, and thus may exhibit idealized noise characteristics and regular class distributions. The UrbanIoT dataset is not peer-reviewed and has not been independently validated against a peer-reviewed ground truth data collection procedure. These factors reduce the external validity of the results reported here and require validation with actual sensor deployments before the clinical and operational deployment of HECIF.

The two-edge and cloud processing environments were modeled by constraining computational resources to a single physical machine rather than deploying HECIF on real distributed machines. Therefore, the communication overhead between the edge and cloud tiers was modeled rather than measured using the IoT Sensor–Cloud Transmission dataset, and the energy consumption analysis was conducted using proxy metrics (normalized CPU load) instead of actual power consumption. Further testing of HECIF should be performed on edge devices such as NVIDIA’s Jetson or Raspberry Pi 4 nodes using a real cloud endpoint.

HECIF was trained and evaluated using a fixed train/test split from the same dataset, without being exposed to data from other hospitals, cities, or network topologies. However, distribution shifts present in real-world deployments can be caused by population shifts, hardware aging, firmware updates, and seasonal changes. The optimal LRI threshold (θ_LRI_ = 0.65) and the optimal FRS temperature parameter (α = 2) were tuned on a validation fold from the same distribution, which may not generalize to other deployment contexts without re-calibration. Federated learning and online adaptation mechanisms are noted as key areas for future research.

While SMOTE helped resolve the class imbalance in the critical patient status class of the medical IoT sensing dataset, oversampling with SMOTE can lead to overfitting the minority class in the training fold if the synthetic samples are not adequately diverse. To prevent the effect of class imbalance on the performance comparison, the MCC metric was chosen. However, it may be necessary to tune the threshold for the reliability-weighted decision layer when HECIF is applied to datasets with greater class imbalance.

## 7. Conclusions and Future Work

This paper introduced a novel nine-layer architecture for robust sensing and data fusion for AI in smart healthcare and urban IoT, called HECIF. HECIF was developed and evaluated based on three complementary publicly available datasets: first, a Multi-Sensor Medical IoT dataset containing medical physiological signals; second, a dataset of multimodal smart city sensor streams called UrbanIoT Anomaly; and third, a dataset of an IoT Sensor–Cloud Data Transmission, which provides empirical edge–cloud communication metadata. The framework features an adaptive offloading controller that relies on a Latency Risk Index, a multimodal fusion layer based on attention to integrate multiple predictions, and a reliability-weighted decision layer that accounts for the reliability of each sensor channel when making the offloading decision.

There are four directions for future work. First, HECIF should be tested on real edge devices like Raspberry Pi, NVIDIA Jetson, or other embedded devices, plugged into real IoT gateways and cloud services. Second, future studies should consider live sensor streams from the healthcare and urban environments to address the challenge of real-world noise, packet loss, device diversity, and distribution shift. Third, privacy-preserving and federated learning mechanisms should be incorporated to allow for model updates to be pushed out across the multiple hospitals and city nodes without having to aggregate raw sensor data centrally. Fourth, knowledge distillation, lightweight neural architectures, and adaptive model compression need to be explored to mitigate the cloud dependency and make the inference at the edge more efficient. In conclusion, HECIF offers a practical system-level architecture for reliability-aware AI sensing, and the system remains to be validated for practical deployment in real edge cloud and IoT deployments.

Future work should include the following priority directions: (i) real-world deployment validation on physical edge hardware and real-time IoT infrastructure to test the performance under real data distribution and hardware variations; (ii) integration of federated learning mechanisms for the ability to update the model across distributed hospital and city nodes while preserving the privacy of raw sensor data; (iii) incorporation of online streaming adaptation techniques to overcome distribution shift caused by sensor drift and varying environmental conditions; and (iv) evaluation of HECIF in low-resource linguistic and clinical contexts to demonstrate its suitability for deployment in lower-income healthcare and urban settings. I believe HECIF will be the basic architecture for a new generation of AI sensing systems across domains, including health and smart cities, that are reliability-aware.

## Figures and Tables

**Table 1:** Summary of datasets used for healthcare sensing, urban anomaly detection, and edge–cloud transmission analysis.

Dataset	Domain	Data Type	Main Features	Target Variable	Main Task	Role in HECIF
Multi-Sensor Medical IoT [26]	Healthcare IoT	Tabular/time-series	Heart rate, SpO_2_, temperature, BP, motion, glucose	Patient status class	Physiological classification	Healthcare sensing branch; edge classifier input
UrbanIoT Anomaly [27]	Smart city	Multimodal tabular	Traffic volume, AQI, noise, humidity, PM2.5, luminance	Anomaly type/severity	Urban anomaly detection	Urban sensing branch; cloud learner input
IoT Sensor–Cloud Transmission [28]	Edge–cloud IoT	Tabular	Packet size, RTT, bandwidth, buffer occupancy, CPU load	Processing location	Offloading decision prediction	Offloading controller; reliability scoring

**Table 2:** Unified enriched feature space used by the proposed HECIF framework.

Unified Feature Category	Healthcare Dataset Contribution	Urban Dataset Contribution	Edge–Cloud Dataset Contribution	Fusion Role	Reliability Role	Final Model Input Representation
Temporal dynamics	Rolling vital sign stats, PSI	AQI trend, anomaly duration	RTT jitter variability	Temporal alignment across modalities	Captures signal degradation over time	Time-windowed feature vector (dim 24)
Spatial/contextual	Patient ward ID, device ID	Sensor node coordinates, zone label	Edge node ID, cloud endpoint	Cross-modality contextual anchoring	Node-level reliability weighting	Contextual embedding vector (dim 8)
Severity/anomaly score	PSI	MUSS	FRS	Unified risk representation	Composite reliability index	Scalar severity triplet (dim 3)
Raw sensor readings	HR, SpO_2_, BP, temperature	PM2.5, AQI, noise, traffic	RTT, packet loss, CPU load	Base feature stream for deep encoder	Raw quality assessment	Concatenated raw vector (dim 42)
Derived binary flags	Hypoxia flag, arrhythmia flag	Pollution spike flag, noise flag	High-latency flag, congestion flag	Hard-decision priors for edge classifier	Threshold-based reliability gate	Binary indicator vector (dim 12)

**Table 3:** Cross-validation performance and statistical comparison between HECIF and the strongest baseline.

Metric	Strongest Baseline or Reference Mean ± SD	HECIF Mean ± SD	Test Statistic	*p* Value
Accuracy	CNN/LSTM: 0.886 ± 0.012	0.921 ± 0.009	6.42	0.0030
F1-score	CNN/LSTM: 0.879 ± 0.014	0.913 ± 0.010	5.91	0.0041
AUC	CNN/LSTM: 0.903 ± 0.011	0.938 ± 0.008	7.18	0.0020
MCC	CNN/LSTM: 0.743 ± 0.018	0.821 ± 0.014	8.36	0.0011
Latency, ms	Cloud only: 142.0 ± 4.8	29.0 ± 1.6	−42.75	<0.001
Throughput, samples/s	Cloud only: 310 ± 18	1150 ± 64	28.92	<0.001

**Table 4:** Ablation comparison of the full HECIF framework and reduced variants.

Model Variant	Reliability Weighting	Adaptive Offloading	HECIF Decision Mechanism	Accuracy	F1-Score	AUC	MCC	Latency, ms
Healthcare only modality	No	No	No	0.824 ± 0.018	0.812 ± 0.016	0.846 ± 0.014	0.701 ± 0.020	41.5 ± 2.3
Urban only modality	No	No	No	0.807 ± 0.020	0.798 ± 0.017	0.832 ± 0.015	0.684 ± 0.023	44.2 ± 2.5
Edge cloud only modality	No	No	No	0.791 ± 0.021	0.783 ± 0.018	0.819 ± 0.017	0.661 ± 0.025	38.4 ± 2.1
Healthcare + Urban fusion	Partial	Partial	Partial	0.872 ± 0.015	0.861 ± 0.013	0.884 ± 0.012	0.754 ± 0.018	52.6 ± 3.2
Simple concatenation fusion	No	Yes	Partial	0.884 ± 0.014	0.873 ± 0.012	0.893 ± 0.011	0.771 ± 0.017	35.8 ± 2.0
Attention-based fusion	No	Yes	Partial	0.902 ± 0.012	0.895 ± 0.011	0.912 ± 0.009	0.798 ± 0.015	33.6 ± 1.8
HECIF without edge intelligence	Yes	Partial	Partial	0.891 ± 0.013	0.884 ± 0.012	0.907 ± 0.010	0.786 ± 0.016	46.8 ± 2.7
HECIF without cloud intelligence	Yes	Partial	Partial	0.886 ± 0.014	0.879 ± 0.013	0.901 ± 0.011	0.779 ± 0.017	31.4 ± 1.9
HECIF without reliability weighting	No	Yes	Yes	0.906 ± 0.011	0.898 ± 0.010	0.912 ± 0.009	0.803 ± 0.014	30.2 ± 1.7
Full HECIF	Yes	Yes	Yes	0.921 ± 0.009	0.913 ± 0.010	0.938 ± 0.008	0.821 ± 0.014	29.0 ± 1.6

## Data Availability

The datasets analyzed in this study are publicly available on Kaggle. The Multi-Sensor Medical IoT dataset [26] is available at https://www.kaggle.com/datasets/programmer3/smart-health-iot-sensor-dataset (accessed on 3 April 2026). The UrbanIoT Anomaly: Multimodal Smart City dataset [27] is available at https://www.kaggle.com/datasets/ziya07/urbaniot-anomaly-multimodal-smart-city-dataset (accessed on 3 April 2026). The IoT Sensor–Cloud Data Transmission dataset [28] is available at https://www.kaggle.com/datasets/ziya07/iot-sensorcloud-data-transmission-dataset (accessed on 3 April 2026). No new dataset was generated in this study.

## Appendix A

This appendix provides the detailed dataset-specific enrichment profiles used in the proposed HECIF framework. To improve the readability of the main manuscript, the detailed tables on healthcare, urban sensing, and edge cloud transmission were moved from the main text to the appendix.

Table A1 summarizes the feature groups enriched from the healthcare dataset. The edge classifier is especially relevant because of the Physiological Stress Index (PSI), a low-dimensional, clinically interpretable summary derived from the data, which can be calculated at the edge nodes in a resource-constrained setting.

**Table A1:** Master data enrichment profile for the healthcare sensing dataset.

Feature Group	Original Features	Enriched Features	Transformation Method	Purpose in HECIF	Expected Effect on Model Learning
Vital signs	Heart rate, SpO_2_, temperature, BP	Rolling mean, std, rate-of-change (10 s window)	Sliding window statistics	Capture physiological dynamics over time	Improves temporal discriminability for arrhythmia and hypoxia detection
Motion/activity	Accelerometer magnitude	Step count, motion energy, posture label	Signal energy + threshold classification	Filter motion artifact from vitals	Reduces false positives from movement noise
Glucose/metabolic	Glucose, SpO_2_	Glucose trend slope, hypoxia risk flag	Linear regression over a 5-sample window	Detects metabolic deterioration trajectories	Enables early warning of glycemic events
Composite index	All vitals	Physiological Stress Index (PSI)	Weighted linear combination of normalized features	Single-dimensional severity ranking for edge classifier	Reduces feature dimensionality while preserving clinical semantics
Sensor quality	Signal-to-noise ratio, missing rate	Sensor Quality Score (SQS)	Normalized composite scoring	Input to the reliability-weighted decision layer	Prevents low-quality readings from dominating predictions

Table A2 presents the urban dataset enrichment profile. The Multimodal Urban Severity Score (MUSS) serves as a fusion-ready composite feature that distils heterogeneous environmental readings into a single risk index, improving the performance of the cloud-tier deep learner on multi-class anomaly classification.

**Table A2:** Master data enrichment profile for the urban sensing dataset.

Feature Group	Original Features	Enriched Features	Transformation Method	Purpose in HECIF	Expected Effect on Model Learning
Traffic	Vehicle count, speed, density	Congestion index, speed deviation, anomaly duration	Z-score normalization and rolling window	Detect sustained traffic anomalies vs. transient spikes	Reduces false alarms from momentary traffic fluctuations
Air quality	PM2.5, PM10, NO_2_, AQI	AQI trend, pollution spike flag, hour-of-day bins	Temporal binning and rate-of-change	Identify pollution events with temporal context	Improves recall for environmental anomalies
Meteorological	Temperature, humidity, wind speed	Humidity–temperature interaction, wind chill index	Feature interaction (product)	Capture compound meteorological anomalies	Enables detection of weather-driven anomaly cascades
Acoustic/noise	Decibel level, frequency band	Noise energy, noise duration flag	Energy computation and binary threshold	Distinguish sustained vs. impulsive acoustic events	Separates construction noise from vehicular incidents
Composite score	All modalities	Multimodal Urban Severity Score (MUSS)	Weighted fusion of normalized modality scores	Single-score urban risk index for cloud learner	Compresses multimodal inputs while preserving anomaly discriminability

Table A3 details the transmission dataset enrichment profile. The Latency Risk Index (LRI) and Fusion Readiness Score (FRS) are the two most consequential engineered features in HECIF: the LRI drives offloading decisions, while the FRS modulates confidence weights in the reliability-weighted fusion layer.

**Table A3:** Master data enrichment profile for the IoT sensor–cloud transmission dataset.

Feature Group	Original Features	Enriched Features	Transformation Method	Purpose in HECIF	Expected Effect on Edge–Cloud Decision Making
Latency/RTT	Round-trip time (RTT), jitter	Latency Risk Index (LRI), jitter variability	RTT normalized to 95th-percentile baseline	Primary input to the offloading threshold controller	Enables latency-aware dynamic routing of inference tasks
Bandwidth	Available bandwidth, utilization rate	Bandwidth headroom, burst utilization ratio	Ratio computation; rolling 5-sample mean	Assess cloud channel capacity for offloading	Prevents offloading during bandwidth congestion
Packet/buffer	Packet loss rate, buffer occupancy	Sensor Quality Score (SQS), effective throughput	Composite normalized scoring	Reliability input: cloud quality gating	Discounts decisions during high-loss transmission periods
Compute	Edge CPU load, cloud server load	Edge suitability score, cloud suitability score	Complementary normalization: 1 − edge_CPU, 1 − cloud_CPU	Adaptive offloading decision	Prevents overloading of edge or cloud nodes
Composite	LRI, SQS, bandwidth headroom	Fusion Readiness Score (FRS)	Harmonic mean of LRI, SQS, and bandwidth headroom	Input to the reliability-weighted decision layer	Provides a single transmission quality signal for weighted fusion
